# Improving the Light-Induced Spin Transition Efficiency in Ni(II)-Based Macrocyclic-Ligand Complexes

**DOI:** 10.3390/molecules24234249

**Published:** 2019-11-22

**Authors:** Alex-Adrian Farcaș, Attila Bende

**Affiliations:** 1 National Institute for Research and Development of Isotopic and Molecular Technologies, Donat Street, No. 67-103, Ro-400293 Cluj-Napoca, Romania; alexfadrian@yahoo.com; 2 Faculty of Physics, “Babeş-Bolyai” University, Mihail Kogalniceanu Street No. 1, Ro-400084 Cluj-Napoca, Romania

**Keywords:** metal-ligand octahedral coordination, intersystem crossing, TD-DFT, singlet-triplet spin transition, spin-orbit coupling

## Abstract

The structural stability and photoabsorption properties of Ni(II)-based metal-organic complexes with octahedral coordination having different planar ligand ring structures were investigated employing density functional theory (DFT) and its time-dependent extension (TD-DFT) considering the M06 exchange-correlation functional and the Def2-TZVP basis set. The results showed that the molecular composition of different planar cyclic ligand structures had significant influences on the structural stability and photoabsorption properties of metal-organic complexes. Only those planar ligands that contained aromatic rings met the basic criteria (thermal stability, structural reversibility, and appropriate excitation frequency domain) for light-induced excited spin state trapping, but their spin transition efficiencies were very different. While, in all three aromatic cases, the singlet electronic excitations induced charge distribution that could help in the singlet-to-triplet spin transition, and triplet excitations, which could assist in the backward (triplet-to-singlet) spin transition, was found only for one complex.

## 1. Introduction

Understanding and controlling the spin-crossover (SCO) properties of metal-organic supramolecular complexes can be of particular interest for potential applications in molecular spintronics [1,2]. The wide range of possibilities for choosing the proper metal ions, organic ligand or the coordination configuration gives us various solutions for designing metal-organic complexes with desired properties through which it is possible to improve the efficiency of the spin crossing, photocatalytic activity, or structural thermal expansion [3,4,5,6]. Apart from the feasibility through chemical synthesis of these newly designed supramolecular complexes, they should present high structural stability and strong efficiency of the low spin-high spin transition to have good and long-lasting reversibility between the spin states. From the energetic stability point of view, based on the energy decomposition study of the ligand bonds at coupled-cluster level of theory, we already observed that structures, based on the diketo-pyrphyrin macrocycle as a planar ligand, showed more stable five- and six-coordination metal-organic complexes than that found for the porphyrin case. Here, unlike the porphyrin, in the diketo-pyrphyrin macrocycle case, the deprotonation occurs not in the plane of the macrocycle but the axial directions, and therefore a much stronger ligand bond is obtained due to the extra electrostatic interaction between the positively charged central metal cation and the negatively charged vertical ligands [7]. On the other hand, the efficiency of the spin transition is mainly influenced by the non-adiabatic coupling between the potential energy surfaces [8,9], as well as by the strength of spin-orbit couplings between the two spin states [8,10,11].

A special class of the SCO materials is defined by the so-called light-induced excited spin state trapping (LIESST) effect, where through the irradiation of the metal-ligand center by selective laser frequencies, one can modulate the physical properties of SCO materials [12,13,14,15,16,17,18]. In this way, the condition of the selective excitation of the electronic structure with different spin states induces a new selectivity criterion over the previous two [19,20,21]. The ground state electronic structures of the metal-organic complexes are mainly defined by the charge transfer effects from the electron reach ligand systems to the central positive metal ions [22,23,24]. Pump-probe experimental investigations of the electronic transitions have shown that metal-to-ligand charge transfer (MLCT) excited states are involved in the high to low spin transition in pseudo-octahedral polypyridal iron coordination complexes [25,26,27,28]. Not only the MLCT effect could be relevant for the spin transition process but also the back transition, the so-called ligand-to-metal charge transfer (LMCT) effects [29,30,31]. Moreover, it was experimentally proved [5] that the ligand substitution could drastically change the excited state relaxation over the MLCT states and influence the efficiency of the spin transition. In general, it can be said that inducing charge transitions at the electronic excited state level and thereby changing the spin state of the system is strongly related to the absorption efficiency of the laser field radiation and, therefore, it is important to understand in more detail the photophysical and photochemical behavior of such complex supramolecular systems.

On the other hand, a detailed theoretical investigation of the electronically excited states of such metal-organic complexes requires particularly large computer capacity due to the needs of the multireference treatment of a large number of atoms and valence electrons [32,33,34]. It has been demonstrated [35,36,37,38,39,40,41,42] that the time-dependent density functional theory (TD-DFT) can be an alternative solution for obtaining accurate geometries, vibrational frequencies, UV absorption spectra, and relative energies of the states involved in the light-induced spin-crossover phenomena.

Spin transitions over the electronically excited states involve complicated relaxation processes, starting from the absorption of laser radiation and followed by the crossings between potential surfaces characteristic for similar (conical intersection) or different (intersystem crossing) spin states [43]. In most of the cases, not just the first or the second excited states are populated through the laser excitation but much higher electronic states [42] and, therefore, is almost unpredictable between what specific electronic states the spin transition will take place. Another very important aspect that also needs to be considered is the problem of the selective excitation of the electronic levels having different spin states. This selectivity can be easily manipulated by a proper choice of ligand structures, which show different photochemical behaviors, and thus one can find favorable excitation frequencies for spin transitions.

In the present work, we explored in more detail the role of different organic ligands in the thermal stability, structural reversibility, as well as the presence of appropriate excitation frequency domain for different Ni(II)-based metal-organic complexes with octahedral coordination. Based on the results, structures with efficient SCO properties from the laser excitation point of view would be proposed.

## 2. Results and Discussions

### 2.1. Structures and Energetics

**Figure 1:**
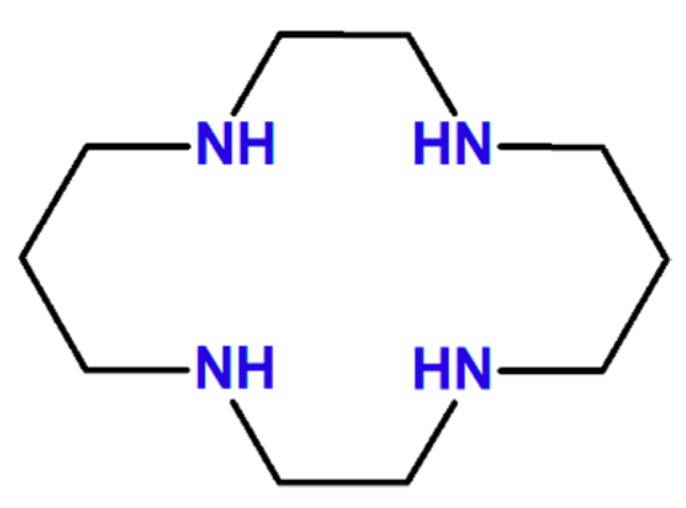
The chemical formula of the cyclam structure.

**Figure 2:**
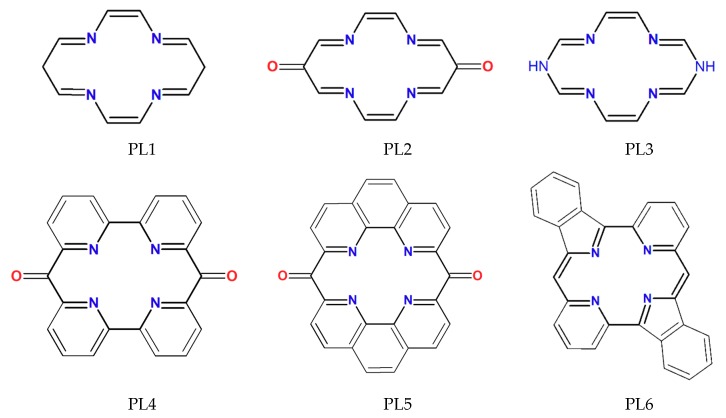
The chemical formulas of the proposed planar ligand (PL) structures for the octahedral coordinated metal-organic complexes.

Based on our preliminary investigation [7], the planar ligand (PL) macrocycle structure having the cyclam-type [11] ring configuration (see Figure 1) showed better spin-state bistability than that found for the porphyrin case. This was especially true for the stability of singlet geometry where the vertical ligands (VL) were much strongly bounded to the Ni(II) ion in the first case than in the second one due to the extra electrostatic interaction between the negatively charged VL and the positively charged central metal ion. Accordingly, different PL macrocycle structures were chosen where the saturated C-C and C-N bonds from the cyclam ring configuration were replaced with double bonds or aromatic fragments (see Figure 2).

**Figure 3:**
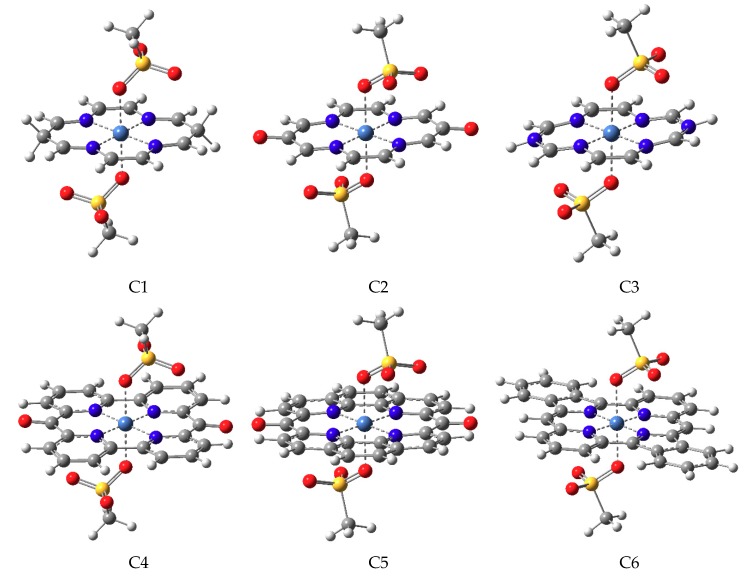
The equilibrium geometry configuration of the six proposed metal-organic complexes (C1–C6) with triplet spin configuration.

The double ionized Ni(II) metal ion was six-coordinated by the lone pair electrons of the four nitrogen atoms of the PL and by the two mesylate anions (CH_3_SO_3_−) as VL, which also neutralized the positive charges of Ni(II). The equilibrium geometries for both singlet and triplet spin configurations, as well as the minimum energy crossing point geometries of the ground state singlet and triplet energies for six different six-coordinated metal-organic complexes (C1–C6), were obtained using the M06/def2-tzvp level of theory. Since the LIESST phenomenon assumed two stable geometry configurations for the singlet and the triplet spin states, it was necessary to have a sufficiently high energy barrier between these two stable geometries, to induce spin transitions in a controlled manner. Accordingly, the geometry of the saddle point is defined as that point on the potential energy hyper-surface where the singlet and triplet electronic energies are identical. This point is nothing else than the so-called minimum energy crossing point (MECP), and in the following, we would characterize the C1–C6 structures by considering the singlet and triplet equilibrium, as well as the MECP geometries. The equilibrium geometry conformations for the triplet spin configuration are shown in Figure 3, while those for the MECP and singlet geometries are collected in Figure S1 of the supporting material (SM) file. Analyzing the geometry configurations obtained for the triplet spin state, all C1–C6 structures geometries showed well-defined octahedral coordination configuration (four N_∥_···Ni planar and two O_⊥_···Ni vertical ligands). The N_∥_···Ni planar ligand bond distances varied from the shortest value of 1.809 Å in the C6 case to the largest ligand bond length of 2.006 Å found for the C2 geometry. In the case of O_⊥_···Ni vertical ligands, the shortest and longest ligand bond values were obtained for the C2 complex (d(O_⊥_···Ni) = 2.106 Å) and the C3 complex (d(O_⊥_···Ni) = 2.164 Å), respectively. In the case of MECP geometries, the octahedral coordination form was still kept, with the difference that the O_⊥_···Ni vertical ligands became larger, while the planar N_∥_···Ni coordination bonds were shorter. However, the vertical and planar ligand bond distances did not vary equally. While the O_⊥_···Ni vertical ligands enlarged, on average, with 0.311 Å, the change in the N_∥_···Ni planar ligands was only 0.065 Å. Geometries with singlet spin states were considered as the most frangible cases since the octahedral coordination stability was much weaker than for the triplet spin states or MECP geometries. The O_⊥_···Ni ligands further enlarged, and the fragment molecular interactions could not keep together the mesylate anions and the metal ion. In this sense, the most striking case was found for C3 geometry, where the mesylate anion fragments moved away from the central metal cation, deprotonated the NH fragment of the PL3 planar cyclic ligand, and formed hydrogen-bonds (CH_3_SO_2_-OH···N) with the nitrogen. In the other five cases, the metal-organic systems retained their octahedral coordination configuration, but the vertical O_⊥_···Ni ligands further enlarged, on average, with 0.2 Å, and the N_∥_···Ni planar ligands shortened with 0.023 Å. Since, through the deformation of the octahedral coordination configuration for the singlet spin state, C3 broke the back and forth transition between the geometries, with singlet and triplet spin states, we excluded the C3 system from our further analysis.

To ensure that thermal fluctuations can also induce geometric transitions, the relative conformational energies for the two minima and the saddle point between them was computed for the five (C1, C2, C4–C6) remained metal-organic systems (see Table 1). All of them showed the well-known asymmetric double-well potential profile. In four cases (C1, C2, C4, and C5), the triplet geometry had the lower energy minima, while for the C6, the singlet geometry showed stronger coordination than its corresponding triplet geometry. As important as the relative position of the two minima, were also the height of the right and left barriers between the minima. In this respect, C1, C2, and C5 systems performed worse than C4 and C6 since, for the first case, the barriers between the singlet energy minima and the MECP were around 0.1 eV or even lower, while, for the C4 case, the singlet minima-MECP barrier was 0.2 eV. For the C6 complex, the reverse case of C4 was observed. Here, the triplet minima-MECP barrier was smaller, but its 0.28 eV value was already high enough to prevent the thermally induced transition. Accordingly, it can be concluded that from the LIESST phenomenon point of view, the C4 and C6 metal-organic complexes were the energetically most stable systems, while, in the C1, C2, and C5 cases, the singlet to triplet spin transition could occur even at the ground state electronic level. However, this transition also depended on the magnitude of the spin-orbit [9] and the non-adiabatic [8,39] couplings between the singlet and triplet spin states of the MECP geometry.

As it was shown before, it might happen that the given metal-organic complex does not show the well-defined bistable character, preferring always only one of the spin states due to the small barrier between the two spin states. The square-planar coordinated Ni(II) metal-organic complex always prefers the singlet spin state, but its higher coordination complex turns over to the triplet spin state. This phenomenon is called coordination-induced spin-state switching (or CISSS) [44,45,46,47]. Due to the small energy difference between the equilibrium geometry for the singlet spin state and the MECP geometry, in the case of C5 (0.086 eV), it might have happened that the bistable character was lost and the system remained only in its triplet spin state.

Since the high spin (HS)-low spin (LS) energetics depend strongly on the amount of HF exchange [48,49,50] considered in our exchange-correlation (XC) functional, as well as it also depends on the nature of the central metal ion [48], it is important to compare our results with those obtained with other XC functionals. Accordingly, six different XC functionals were considered, and their adiabatic singlet-triplet energy gaps were compared (see Table S1 from the Supplementary Materials file). All six cases gave the triplet spin state geometry as the lowest electronic energy configuration, while the relative deviation of the adiabatic energy gap was less than 0.3 eV. It is also known that external perturbations like thermal effects can considerably change the adiabatic energy gap calculated at 0 K by the so-called enthalpy−entropy compensation effects [50,51]. The normal mode vibrational analysis was performed using the M06 XC functional for both the low- and high-spin cases, and the thermally corrected adiabatic energy gap was computed. The reaction enthalpy effects enlarged the gap with about 0.53 eV, while the entropy effects were almost negligible (reduced the gap with about 0.006 eV). For the M06 functional, they finally gave an adiabatic LS-HS gap of 0.97 eV.

It has also been observed that the nature of the solvent can seriously influence the SCO characteristics of metal-organic supramolecular complexes [52]. Accordingly, two solvents (chloroform and DMSO) with different solvent properties were considered in order to follow the influence of the environment on the adiabatic HS-LS energy gap. The characteristic ligand bond distances and conformational energy differences for the singlet and triplet spin configurations, as well as for the MECP geometries calculated in non-polar (chloroform, ε = 4.8) and aprotic polar (DMSO, ε = 47.2) solvent environments, are given in Table S2 of the Supplementary Materials file. The results obtained at M06/CPCM/def2-TZVP level of theory showed that the influence of the solvent environments revealed a reduction of the adiabatic HS-LS energy gap, from 0.448 eV in vacuum to 0.380 eV in chloroform and 0.308 eV in DMSO, respectively. The energy barrier defined by the MECP geometry also decreased from the 0.642 eV in vacuum to 0.434 eV in chloroform and 0.527 eV in DMSO, respectively, as compared to the reference energy level of the triplet spin state. In summary, it could be said that solvent effects did not substantially change the relative positions of the HS-LS energy levels, they shifted down a bit the energies both for the singlet spin state and for the barrier between the singlet and triplet spin states as compared to the reference energy level.

The problem of reversibility and stability also includes the ability of the metal-organic complex to form octahedral coordination configuration. Accordingly, the thermodynamical condition for forming the metal-organic complex with octahedral coordination configuration for the C4 case was studied at M06/def2-tzvp level of theory. Once the macrocycle of diketo-pyrphyrin is formed, the central metal is bounded by the lone pair electrons of the macrocycle’s nitrogen atoms in the form of metal dibromide salt [53,54], building a square planar coordination complex. In order to form octahedral coordination, the bromide ligands need to be reduced through the deprotonation reaction of the mesylate groups. The reaction enthalpy of this process is −26.75 kcal/mol, while the Gibbs free energy of the reaction is −19.69 kcal/mol at 0 °C and −21.76 kcal/mol at −80 °C, respectively. The negative energy values indicate to us that the formation of the octahedral coordination configuration is thermodynamically feasible. From the octahedral coordination formation point of view, not only the thermodynamics is important but also the reaction kinetics. For a detailed study of this aspect, one needs to perform large scale calculations to find the reaction path described by the intrinsic reaction coordinates, which is quite far from our goal of study. However, to get an idea of how the square-plane coordinative system can bind the mesylate groups, we built a complex where two neutral mesylate groups were axially attached to the metal bounded diketo-pyrphyrin. The intermolecular interaction between the Ni(II)–diketo-pyrphyrin and the two neutral mesylate groups was −42.99 kcal/mol obtained at M06/def2-tzvp level of theory, and its geometry is shown in Figure S2 of the Supplementary Materials file. The shortest Ni···O bond distances were 2.715 and 3.032 Å, respectively. The interaction between the metal bounded cyclic-ligand planar complex and the axial ligands became much stronger if the OH fragments of the mesylate groups were deprotonated. Accordingly, the intermolecular interaction between the Ni(II)–diketo-pyrphyrin and the two neutral mesylate groups was −311.22 kcal/mol obtained at the same theory level of M06/def2-tzvp.

### 2.2. Singlet and Triplet Electronic Transitions

**Figure 4:**
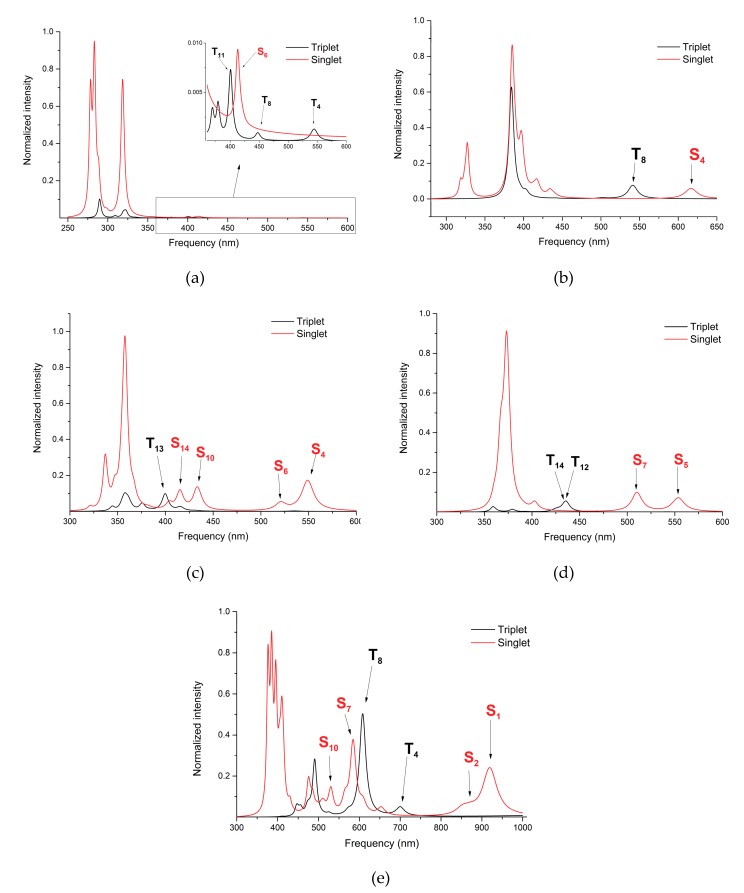
The UV absorption spectra calculated for the singlet and triplet spin states of the (**a**) C1, (**b**) C2, (**c**) C4, (**d**) C5, and (**e**) C6 metal-organic complexes obtained at TD-DFT/M06/Def2-TZVP level of theory. TD-DFT, time-dependent density functional theory.

Although the conformational energy analysis did not fully recommend the further investigation of the C1, C2, and C5 complexes, they were still kept to find a correlation between the extent of electron delocalization over the unsaturated bonds or aromatic rings and the electronic excitation. The UV absorption spectra for the C1, C2, C4–C6 complexes, including the first 30 electronic excited state levels, with both the singlet and triplet spin configurations, were computed considering the TD-DFT method using the same M06 XC functional and Def2-TZVP basis set. Their spectral profiles are shown in Figure 4.

In the case of the singlet spin state, the UV absorption spectra perturbed by the close-lying triplet excited electronic states through the spin-orbit coupling (SOC) was also computed [55]. Analyzing the spectral profile presented in Figure 4a, the theoretical UV spectra computed for the C1 structure showed very poor spectral selectivity for the singlet and the triplet excitations. This was because, on the one hand, the absorption intensity in the spectral range of 350–600 nm was extremely weak, and on the other hand, the singlet and the triplet spectral profiles strongly overlapped, and thus their selectivity became poor. The spectral profile for the C2 structure showed a bit better selectivity behavior. For this case, it was found one excitation frequency for each spin state with relatively low intensities: S_4_ (616 nm) for singlet and T_8_ (541 nm) for triplet. Structures C1 and C2 were built based on the PL1 and PL2 planar ligands, which contained unsaturated C=C or C=O bonds but not aromatic rings. It seems that by simply considering unsaturated double bonds, the absorption properties could not be significantly improved, in the sense that one could obtain good selectivity for singlet and triplet excitations. However, it should also be noted that the presence of C=O as a fragment in the planar cyclic ligand structure could provide encouraging results as regards the selective excitation of the two spin states. Based on the results regarding the spin selectivity of the UV excitation corroborated with the energetical finding for singlet and triplet equilibrium geometries, as well as the energy barrier between them defined by the MECP geometry, one could conclude that C1 and C2 metal-organic systems are not suitable as laser-controlled spin-crossover materials.

The planar ligand geometries of the proposed C4–C6 metal-organic systems contained a different type of heteroaromatic rings, starting from the simple case of bipyridine (C4) and followed by the more complex 1,10-phenanthroline (C5) and 2-pyridyl-isoindole (C6) structures. Considering these heteroaromatic rings, a more intense band in the UV region of the absorption spectra was obtained for the proposed C4 and C5 metal-organics. In almost all cases of singlet and triplet electronic excitations, at least two electronic excited states could be considered for initiating LIESST transition. Accordingly, the relevant excited states for the C4 were S_4_ (549 nm), S_6_ (520 nm), S_10_ (436 nm), and S_14_ (415 nm), as well as T_13_ (400 nm), for singlet and triplet spin, respectively. For C5, they were S_5_ (554 nm) and S_7_ (510 nm), as well as T_12_ (436 nm) and T_14_ (429 nm), respectively, while those for C6 were S_1_ (920 nm), S_2_ (871 nm), S_7_ (586 nm), and S_10_ (530 nm), as well as T_4_ (700 nm) and T_8_ (608 nm). The most relevant electronic excited states and their oscillator strength for the five proposed (C1, C2, C4–C6) metal-organic complexes are given in Table 2.

**Figure 5:**
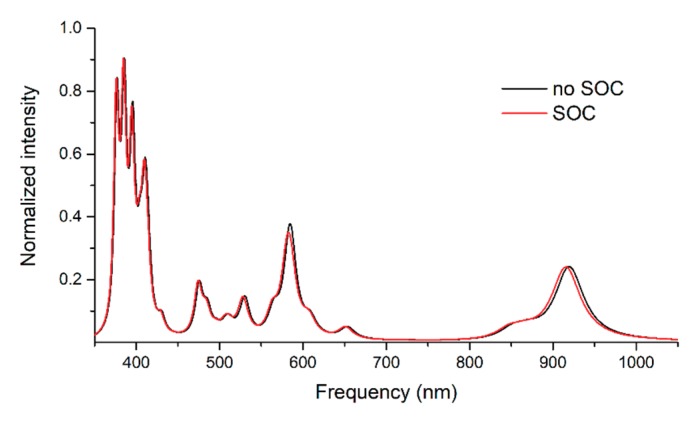
The theoretical UV absorption spectra calculated with and without spin-orbit effects for the singlet spin configuration of the C6 equilibrium geometry at TD-DFT/M06/def2-TZVP level of theory.

It was demonstrated that the spin-orbit effects could influence the shape of the UV absorption spectra [56] by splitting spectral lines characteristic for different electronic transitions. Since the UV spectra of the C6 has the most absorption peaks in the spectral domain of 400–1000 nm, the spin-orbit coupling correction was applied to this case as control calculation. Comparing the two spectral shapes, shown in Figure 5, one could observe that the SOC effects did not induce a significant change in the spectral shape, and only a small blueshift of 2–4 nm was found.

However, the presence of proper excitation frequencies for the singlet and triplet electronic states is not an unequivocal condition for inducing a spin transition. We also needed to analyze what types of electron transitions were induced by the laser excitations and how efficiently could these excitations promote the formation of an electron distribution, which is close to the other spin state electron configuration. But first, we analyzed the difference between singlet and triplet ground-state electron configurations from the electron population point of view. The NPA broken down into molecular fragments (central metal ion, cyclic planar ligand, and vertical ligands) showed the following fractional electron charge arrangement. In the case of the C4 complex with a singlet spin state, the fractional charge population of the double ionized central nickel ion was +0.61 e, that of the cyclic planar ligand was +1.13 e, while −0.87 e could be found on each mesylate anion. This means that an amount of 1.39 e charges were attracted by the Ni(II) ion, 1.13 e from the planar cyclic ligand, and 0.13 e from each mesylate anions. The natural electron configuration of the central Ni(II) atom was Ar4s[0.26]3d[8.66]4p[0.45]4d[0.02], while the 3d orbital population were $d_{xy}$ [1.50], $d_{xz}$ [1.85], $d_{yz}$ [1.85], $d_{x^{2}-y^{2}}$ [1.57], and $d_{z^{2}}$ [1.92]. The corresponding fractional charge population of C4 with triplet spin configuration was 0.81 e for the Ni(II) ion, 0.74 e for the cyclic planar ligand, and −0.78 e for each mesylate anion. Accordingly, the amount of charge transfer from the ligands to the Ni(II) ion was 1.19 e, 0.74 e from the cyclic planar, and 0.44 e from the two vertical ligands. The natural charge population of the central Ni(II) atom was Ar4s[α:0.12,β:0.12]3d[α:4.95,β:3.37]4p[α:0.27,β:0.29]4d[α:0.02,β:0.01], while the 3d orbital population were $d_{xy}$ [α:0.99, β:0.79], $d_{xz}$ [α:0.99, β:0.48], $d_{yz}$ [α:0.99, β:0.93], $d_{x^{2}-y^{2}}$ [α:0.99, β:0.57], and $d_{z^{2}}$ [α:0.99, β:0.59]. In both spin configuration cases, there were significant charge transfers from the ligand fragments (planar and vertical) to the central Ni(II) cation. Comparing the charge transfer effects for the singlet and the triplet spin configurations, one could conclude that, in the singlet case, the Ni(II) metal cation attracted more electron charges from the ligands than that found for the triplet case. At the same time, one should also mention that, in the case of the triplet spin state, the amount of charges transferred from the vertical ligands to the Ni(II) cation was almost double than that obtained for the singlet spin case, while the number of charges transferred from the planar ligands to the Ni(II) cation was significantly smaller than that found for the singlet spin case. Similar behaviors were found for C5 and the C6 structures as regards their charge distribution (see Table S3 from the Supplementary Materials file).

Knowing the charge distribution at ground state electronic level, broken down into details over the constituent fragments (ligands and metal cation), it would be interesting to analyze how the laser excitation would change the ground state charge density. Accordingly, the Natural Difference Orbitals (NDO), defined as the difference between the ground state and the given electronic excited state orbital densities, were computed for each relevant excited state of C1, C2, C4–C6 complexes. Their graphics are shown in Table S4 of the Supplementary Materials file. As we already have seen, the UV spectral profile analysis pointed out that only the C4–C6 proposed geometries would be appropriate for initiating LIESST transitions. Therefore, the analysis of the NDOs would be applied only to these three complexes. For the singlet electronic states of the C4 complex, the lowest—and with reasonably good—efficiency of excitation was obtained for the S_4_ excited state. The NDO orbital profile of the S_0_ → S_4_ excitation showed an electron charge redistribution from the central metal cation ($3d_{z^{2}}$ orbital depopulation) and partially from the vertical ligands to the parallel cyclic ligands (see Table S4 in the Supplementary Materials file with NDO orbitals for C4, “hole”—orange and “electron” with blue colors). This represented the MLCT type excitation. The atomic orbital occupancies (holes and electrons of the NDO) of the electron population for the S_4_ excited state suggested us that the formation of the triplet spin configuration would occur much easier after the S_0_ → S_4_ excitation since its electron distribution was more like the one found for the triplet than for the ground state singlet. Of course, the spin transition is not as simple, it highly depends on the spin-orbit [9] and the non-adiabatic [8,39] couplings between the singlet and triplet electronic excited states and, therefore, a “static picture” of their energetics and electron population are not able to tell us exactly what electronically excited states are particularly involved in the spin flipping. It requires a real-time quantum dynamics simulation on these excited state hyper-surfaces [57,58] where the non-adiabatic and vibronic coupling terms close to the crossings are also computed. On the other hand, this “static” picture is more confusing for triplets because no excitation could be found, which would promote an electron redistribution favorable toward the electron configuration with a singlet spin state. T_13_, the lowest, most intense triplet excited state showed a similar excitation mechanism than that obtained for S_4_. Namely, it induced charge transfer from the Ni(II) ($3d_{z^{2}}$ orbital depopulation) and mesylate fragments to the planar cyclic ligands. Similar behavior was found for the C5 case, both with singlet and triplet spin configurations. For the C4 and C5 complexes, in the case of triplet excitations, the “static” picture did not give any indication as to whether the triplet excitation would help a spin transition or not. To elucidate the role of the triplet excitation in the triplet-to-singlet spin transition, again, one needs to perform real-time quantum dynamics simulation on these excited state hyper-surfaces [57,58]. Regarding the C6 metal-organic complex, the electron rearrangement induced by the excitations showed different mechanisms. First, in the singlet spin case, the symmetry of the vertical ligands was broken and, therefore, in the case of S_0_ → S_1_ excitation, only the central metal cation (with $3d_{z^{2}}$ orbital depopulation) and one of the mesylate fragments were those molecular units where the electron holes were located, while the excited electrons were spread over on the planar ligand. This electron transition clearly showed an MLCT character. Second, in the case of the triplet excitation, the T_0_ → T_8_ transition showed a combined ligand-to-ligand and ligand-to-metal characters, where the first type was mainly local, while the last was charge-transfer excitation. This LMCT-type excitation, through its charge redistribution, could support the triplet-to-singlet spin transition, which was not observed in the C4 and C5 cases.

## 3. Materials and Methods

The equilibrium geometries considering both the low (singlet) and high (triplet) spin states were optimized at density functional level of theory, including the M06 [59] exchange-correlation functional and the def2-TZVP [60] basis set as implemented in the Orca [61,62] program suite. M06 is a global hybrid functional with 27% Hartree-Fock (HF) exchange, developed mainly for main group thermochemistry and non-covalent interactions, transition metal thermochemistry, as well as metal-organics. For the latter case, the good performance of the M06 XC functional was reported by Zhao et al. [63] and Delcey et al. [64]. The electronically excited states with low- and high-spin configurations were computed at TD-DFT [65,66,67] level of theory, where, again, M06 functions showed relatively good performance [40,68,69] for both singlet and triplet electronic excited states. The computation of the spin-orbit couplings (SOC) between the singlet and triplet spin states was performed, employing the coupled perturbed Kohn–Sham theory method [70] implemented in the same Orca package. The charge distribution analysis was performed based on the natural bond order (NBO) theory [71,72] using the program module [73] built in the gaussian09 package [74], while the molecular graphics (figures) were created using the Gaussview [75] molecular editor and visualizer software. Solvent effects were taken into account considering the conductor-like polarizable continuum model (C-PCM) [76] implemented in the same Orca package.

## 4. Conclusions

In this article, we investigated the role of the molecular architecture of different organic ligands in the geometrical stability, structural reversibility, and the presence of appropriate excitation frequency domain for different Ni(II)-based metal-organic complexes with octahedral coordination.

It was shown that octahedral coordinated metal-organic complexes based on cyclam-like neutral planar ligands and mesylate anions as axial ligands were energetical feasible, and some of them could present spin-crossover properties, depending on the nature of the planar ligands. It was also demonstrated that the unsaturated bonds and aromatic fragments of the cyclam-like planar cyclic ligands could significantly influence the photophysical and photochemical properties of the metal-organic complexes, namely, (i) define the potential energy surface profile of the equilibrium geometries for the two spin states, as well as the potential barrier between them, given by the minimum energy crossing point of the two spin states; (ii) they have individual UV absorption spectral profiles with characteristic absorption peaks, and based on them, their excitation by laser irradiation using appropriate laser frequencies might be possible; (iii) the electronic charge distribution of their singlet and triplet excited states may assist in inducing much easier different spin transitions, like singlet to triplet or vice versa. A very important question that needs to be answered after these molecular design studies are whether or not their chemical synthesis is achievable. It has already been shown that the chemical syntheses [53,54] of copper- or cobalt-coordinated diketo-pyrphyrin metal-organic complexes are experimentally feasible. Based on these receipts, we considered that the C4 or even the C5 metal-organic complexes could be experimentally synthesized, and their spin-crossover behavior can be studied by spectroscopic techniques.

## Figures and Tables

**Table 1:** The characteristic ligand bond distances (in Å) between the Ni(II) central atom and the oxygen or nitrogen atoms for singlet and triplet spin configurations, as well as for the minimum energy crossing point (MECP) geometries of the ground state singlet and triplet energies of the six proposed metal-organic complexes (C1–C6) obtained at M06/def2-TZVP level of theory. The relative conformational energies (E^conf^ in eV) between singlet, triplet, and MECP geometries are also given in the 5th and 10th columns.

Conf.	Geom.	O_⊥_···Ni (Å)	N_∥_···Ni (Å)	E^conf^ (eV)	Conf.	Geom.	O_⊥_···Ni (Å)	N_∥_···N (Å)	E^conf^ (eV)
C1	Sing.	2.709 2.709	1.890, 1.890 1.889, 1.889	0.329	C2	Sing.	2.654 2.654	1.902, 1.902 1.902, 1.902	0.587
	MECP	2.461 2.460	1.923, 1.923 1.921, 1.921	0.432		MECP	2.461 2.461	1.921, 1.921 1.920, 1.920	0.616
	Trip.	2.127 2.127	1.994, 1.995 1.995, 1.995	0.000		Trip.	2.106 2.106	2.006, 2.006 2.003, 2.003	0.000
C3	Sing.	-	1.851, 1.850 1.850, 1.850	-	C4	Sing.	2.573 2.566	1.921, 1.921 1.915, 1.915	0.448
	MECP	2.459 2.459	1.918, 1.918 1.917, 1.917	-		MECP	2.439 2.438	1.937, 1.937 1.940, 1.940	0.642
	Trip.	2.164 2.164	1.979, 1.979 1.979, 1.979	-		Trip.	2.130 2.130	1.997, 1.997 2.001, 2.001	0.000
C5	Sing.	2.636 2.635	1.904, 1.904 1.902, 1.902	0.479	C6	Sing.	2.466 2.912	1.802, 1.803 1.893, 1.894	0.000
	MECP	2.403 2.403	1.929, 1.929 1.931, 1.931	0.565		MECP	2.236 2.240	1.817, 1.817 1.907, 1.907	0.474
	Trip.	2.140 2.140	1.978, 1.978 1.983, 1.983	0.000		Trip.	2.131 2.133	1.809, 1.810 1.895, 1.896	0.193

**Table 2:** The most relevant electronic excited states (wavenumber given in parenthesis in nm) and their oscillator strength (f^osc^) of the five proposed metal-organic complexes (C1, C2, C4–C6) obtained at TD-DFT/M06/def2-TZVP level of theory, both with singlet (S) and triplet (T) spin configuration.

Conf.	S *	f^osc^	T **	f^osc^	Conf.	S	f^osc^	T	f^osc^
C1	S_6_ (413)	0.0010	T_4_ (545)	0.0001	C2	S_4_ (617)	0.0099	T_8_ (541)	0.0129
	S_10_ (354)	0.0002	T_8_ (448)	0.0001		S_8_ (440)	0.0017	T_14_ (502)	0.0007
	S_14_ (319)	0.1002	T_11_ (401)	0.0009		S_11_ (417)	0.0122	T_15_ (495)	0.0001
C4	**S_4_** (549)	0.0081	T_4_ (535)	0.0001	C5	S_5_ (554)	0.0071	T_6_ (542)	0.0001
	S_6_ (520)	0.0016	T_9_ (431)	0.0001		**S_7_** (510)	0.0099	**T_12_** (436)	0.0054
	S_10_ (436)	0.0016	**T_13_** (400)	0.0046		S_12_ (403)	0.0037	T_14_ (429)	0.0005
	S_14_ (415)	0.0036							
C6	**S_1_** (920)	0.0159	T_4_ (700)	0.0029					
	S_2_ (852)	0.0020	**T_8_** (608)	0.0340					
	S_5_ (654)	0.0026	T_9_ (573)	0.0012					
	S_7_ (586)	0.0055	T_15_ (490)	0.0115					
	S_10_ (530)	0.0081	T_16_ (490)	0.0071					

* S_n_, electronic excited level with singlet spin state, n: order of energy level; ** T_n_, electronic excited level with triplet spin state, n: order of energy level.

## Supplementary Materials

The following are available online at https://www.mdpi.com/1420-3049/24/23/4249/s1, Figure S1: The equilibrium geometry conformation of the six proposed C1–C6 metal-organic complexes for the singlet and triplet spin configurations, as well as for the minimum energy crossing point (MECP) geometries of the ground state singlet and triplet energies calculated at M06/def2-TZVP level of theory, Figure S2: The geometry configuration of the complex built by the Ni(II)–diketo-pyrphyrin and two neutral mesylate groups computed for the singlet spin state at M06/def2-tzvp level of theory, Table S1: The singlet-triplet energy gap (in eV) for the C4 organometallic complex obtained with six different exchange-correlation functionals (M06L, revM06L, B3LYP*, B3LYP*-D3, M06, and MN12-SX) implemented in the ORCA program package and considering the Def2-TZVP basis set, Table S2: The characteristic ligand bond distances (in Å) between the Ni(II) central atom and the oxygen or nitrogen atoms for singlet and triplet spin configurations, as well as for the minimum energy crossing point (MECP) geometries of the ground state singlet and triplet energies of the C4 metal-organic complex obtained at M06/CPCM/def2-TZVP level of theory considering two different solvents. The relative conformational energies (Econf in eV) between singlet, triplet, and MECP geometries are also given in the last column, Table S3: The fractional electron population of different fragments (Ni(II), PL—planar ligand, VL—vertical ligand), the natural electron population of the central Ni cation, as well as the natural electron population of the 3d orbitals of the central Ni cation for the C4–C6 metal-organic complexes, obtained at NBO/M06/def2-TZVP level of theory, Table S4: The orbital shapes of the Natural Difference Orbitals (NDO) obtained as the difference between the corresponding ground state and the electronic excited state densities for the five (C1, C2, C4–C6) metal-organic complexes with octahedral coordination computed at TD-DFT/M06/Def2-TZVP level of theory (blue is the positive, and orange means the negative densities).
