# Supplementary material for: Improving the Light-Induced Spin Transition Efficiency in Ni(II)-Based Macrocyclic-Ligand Complexes
Source: Molecules. 2019 Nov 22;24(23):4249. doi: 10.3390/molecules24234249 (PMC6930591; doi:10.3390/molecules24234249)
Supplement: Supplementary file 1 [file molecules-24-04249-s001.pdf]

Supporting Material

Improving the light-induced spin transition efficiency in Ni(II)-based macrocyclic-ligand complexes

Alex A. Farcaş[a,b] and Attila Bende*,[a]

[a]National Institute for Research and Development of Isotopic and Molecular Technologies, Donat Street, No. 67-103, Ro-400293, Cluj-Napoca, Romania.

[b]Faculty of Physics, "Babeş-Bolyai" University, Mihail Kogalniceanu Street No. 1, Ro-400084 Cluj-Napoca, Romania.

Conf.	Geometry		
	Singlet	MECP	Triplet
**C1**	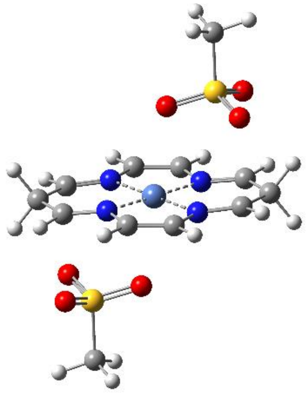	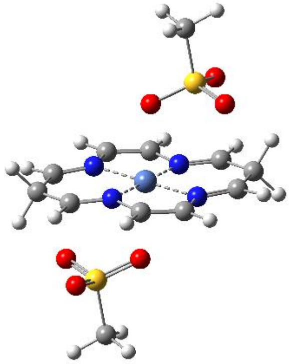	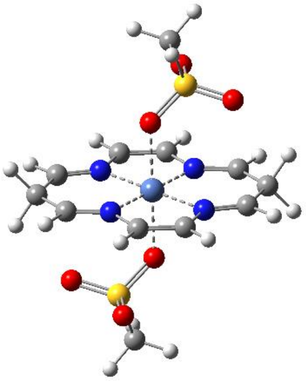
**C2**	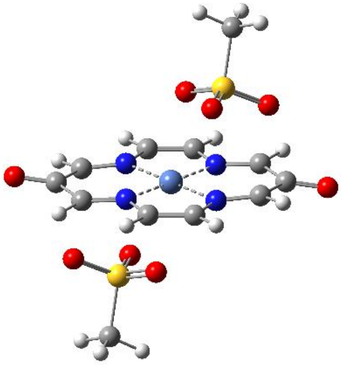	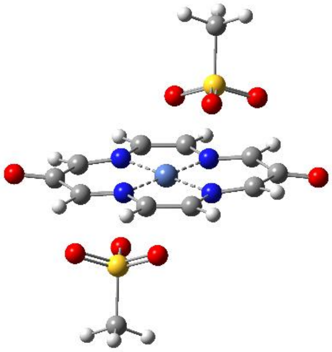	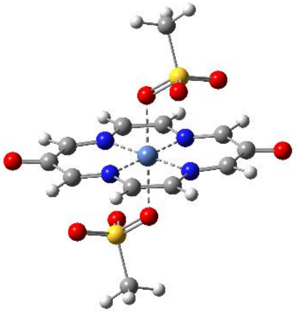
**C3**	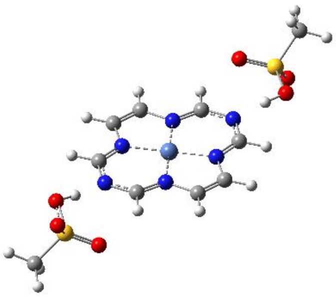	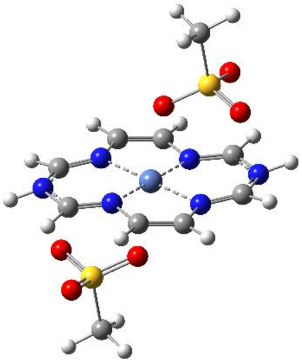	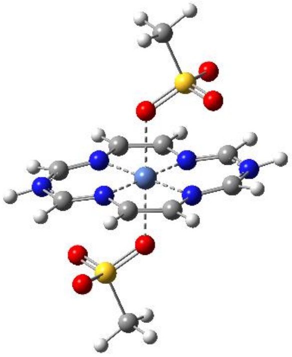

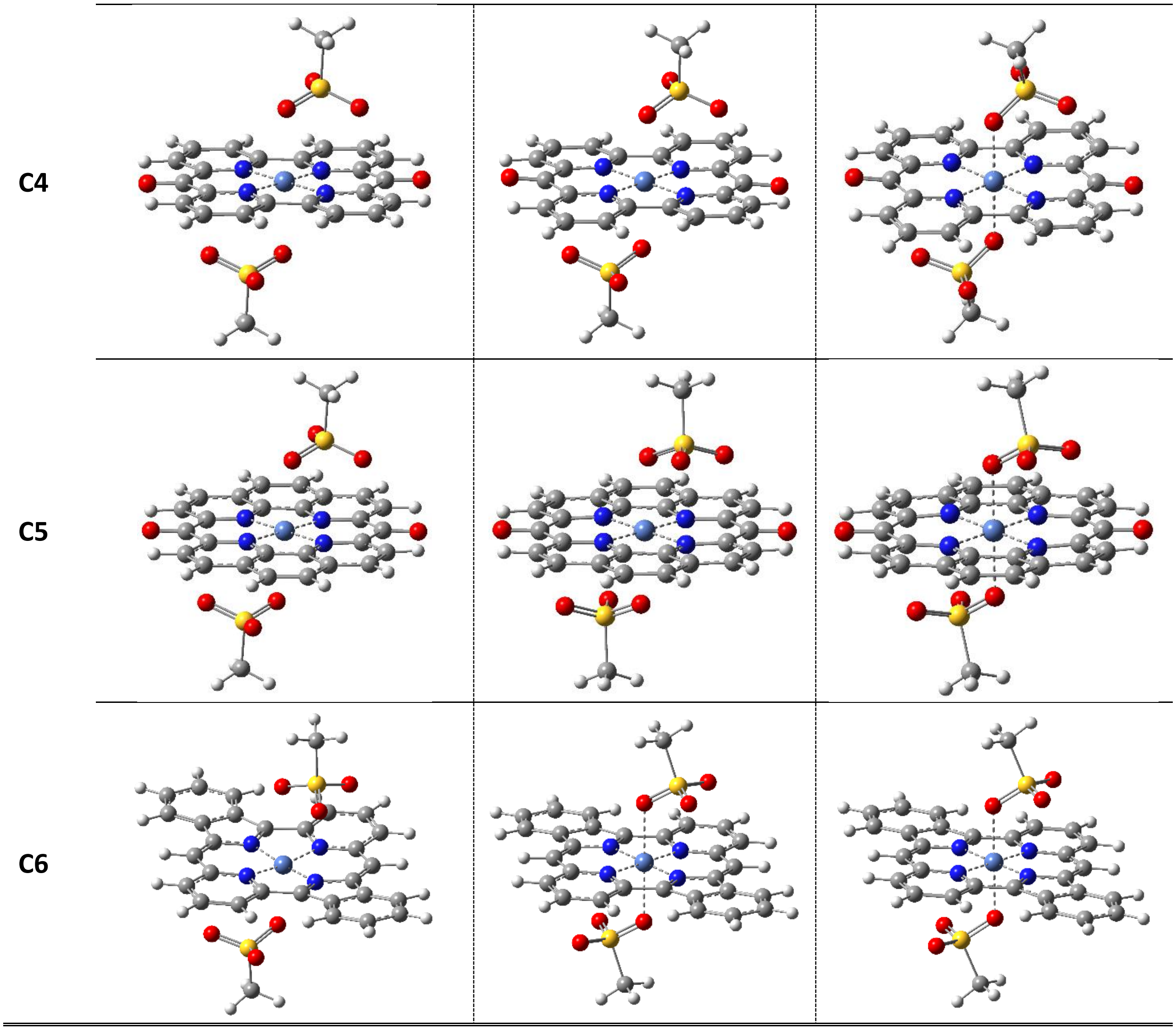

Figure S1. The equilibrium geometry conformation of the six proposed C1-C6 organometallic complexes for the singlet and triplet spin configurations as well as for the minimum energy crossing point (MECP) geometries of the ground state singlet and triplet energies calculated at M06/def2-TZVP level of theory.

Table S1. The singlet-triplet energy gap (in eV) for the C4 organometallic complex obtained with six different exchange-correlation functionals (M06L [1], revM06L [2], B3LYP* [3], B3LYP*-D3 [3-5], M06 [6] and MN12-SX [7]) implemented in the ORCA program package [8,9] and considering the Def2-TZVP basis set [10].

Geom.	XC Functionals					
	M06L	revM06L	B3LYP*	B3LYP*-D3	M06	MN12-SX*
Singlet	0.668	0.587	0.595	0.592	0.448	0.378
MECP	0.722	0.690	0.614	0.606	0.642	N/A
Triplet	0.000	0.000	0.000	0.000	0.000	0.000

*The MN12-SX results were obtained using the GAUSSIAN09 software package [11].

Table S2. The characteristic ligand bond distances (in Å) between the Ni(II) central atom and the oxygen or nitrogen atoms for singlet and triplet spin configurations as well as for the minimum energy crossing point (MECP) geometries of the ground state singlet and triplet energies of the C4 metal-organic complex obtained at M06/CPCM/def2-TZVP level of theory considering two different solvents. The relative conformational energies (E^{conf} in eV) between singlet, triplet, and MECP geometries are also given in the last column.

**Solv.**	**Geom.**	**$O_{\perp}\cdots Ni$ (Å)**	**$N_{\parallel}\cdots Ni$ (Å)**	**E^{conf} (eV)**
Vacuum	Sing.	2.573<br>2.566	1.921, 1.921<br>1.915, 1.915	0.448
	MECP	2.439<br>2.438	1.937, 1.937<br>1.940, 1.940	0.642
	Trip.	2.130<br>2.130	1.997, 1.997<br>2.001, 2.001	0.000
Chloroform	Sing.	2.689<br>2.692	1.912, 1.912<br>1.913, 1.913	0.380
	MECP	2.499<br>2.499	1.934, 1.934<br>1.936, 1.936	0.434
	Trip.	2.159<br>2.159	1.993, 1.993<br>1.994, 1.994	0.000
DMSO	Sing.	2.707<br>2.714	1.911, 1.911<br>1.912, 1.912	0.305
	MECP	2.403<br>2.403	1.929, 1.929<br>1.931, 1.931	0.527
	Trip.	2.165<br>2.166	1.991, 1.992<br>1.993, 1.993	0.000

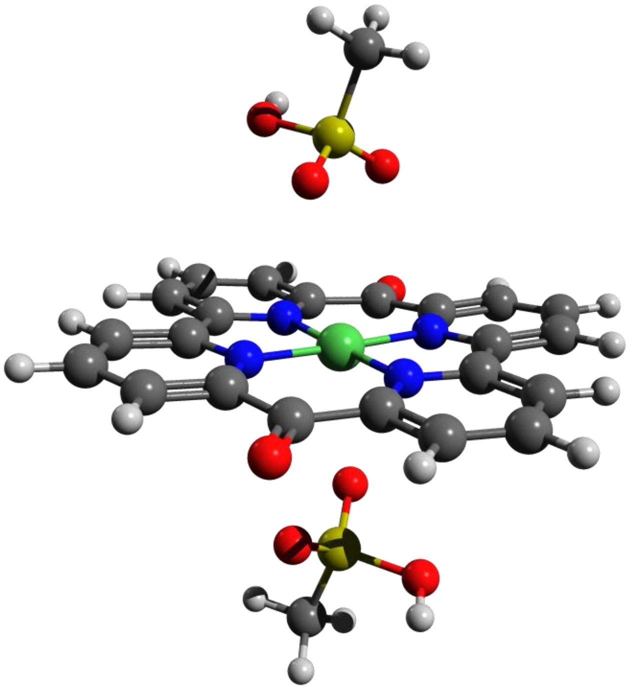

Figure S2. The geometry configuration of the complex built by the Ni(II)–diketo-pyrphyrin and two neutral mesylate groups computed for the singlet spin state at M06/def2-tzvp level of theory.

Table S3. The fractional electron population of different fragments (Ni(II), PL – planar ligand, VL – vertical ligand), the natural electron population of the central Ni cation, as well as the natural electron population of the 3d orbitals of the central Ni cation for the C4-C6 organometallic complexes, obtained at NBO/M06/def2-TZVP level of theory.

**Natural Population of the metal ion and ligand fragments**					
Struct.	$POP^{Ni(II)}$	POP^{PL}	POP^{VL}	CT PL→Ni(II)	CT VL→Ni(II)
$C4^S$	+0.61e	+1.13e	-0.87e<br>-0.87e	-1.13e	-0.13e<br>-0.13e
$C4^T$	+0.81e	+0.74e	-0.78e<br>-0.78e	-0.74e	-0.22e<br>-0.22e
$C5^S$	+0.61e	+1.13e	-0.87e<br>-0.87e	-1.13e	-0.13e<br>-0.13e
$C5^T$	+0.80e	+0.75e	-0.77e<br>-0.77e	-0.75e	-0.23e<br>-0.23e
$C6^S$	+0.54e	+1.25e	-0.87e<br>-0.92e	-1.25e	-0.13e<br>-0.08e
$C6^T$	+0.66e	+0.81e	-0.74e<br>-0.74e	-0.81e	-0.26e<br>-0.26e
**Ni electron population**					
$C4^S$	*Ar*4s[0.26]3d[8.66]4p[0.45]4d[0.02]				
$C4^T$	*Ar*4s[α:0.12,β:0.12]3d[α:4.95,β:3.41]4p[α:0.27,β:0.28]4d[α:0.02,β:0.01]				
$C5^S$	*Ar*4s[0.26]3d[8.68]4p[0.44]4d[0.02]				
$C5^T$	*Ar*4s[α:0.12,β:0.12]3d[α:4.95,β:3.42]4p[α:0.27,β:0.29]4d[α: 0.02,β:0.01]				
$C6^S$	*Ar*4s[0.27]3d[8.69]4p[0.48]4d[0.02]				
$C6^T$	*Ar*4s[α:0.13,β:0.13]3d[α:4.55,β:3.82]4p[α:0.33,β:0.34]4d[α:0.02,β:0.01]				
	**Ni 3d orbital population**				
	d_{xy}	d_{xz}	d_{yz}	$d_{x^2-y^2}$	d_{z^2}
$C4^S$	1.50	1.85	1.85	1.57	1.92
$C4^T$	1.79<br>α:0.99,β:0.80	1.48<br>α:0.99,β:0.49	1.92<br>α:0.98,β:0.94	1.57<br>α:0.99,β:0.58	1.59<br>α:0.99,β:0.60
$C5^S$	1.65	1.68	1.94	1.44	1.96
$C5^T$	1.55<br>α:0.99,β:0.56	1.65<br>α:0.99,β:0.66	1.78<br>α:0.99,β:0.77	1.81<br>α:0.99,β:0.82	1.59<br>α:0.99,β:0.60
$C6^S$	1.93	1.87	1.76	1.29	1.84
$C6^T$	1.95<br>α:0.98,β:0.97	1.91<br>α:0.96,β:0.95	1.45<br>α:0.94,β:0.51	1.34<br>α:0.70,β:0.64	1.71<br>α:0.96,β:0.75

Table S4. The orbital shapes of the Natural Difference Orbitals (NDO) obtained as the difference between the corresponding ground state and the electronic excited state densities for the five (C1, C2, C4-C6) organometallic complexes with octahedral coordination computed at TD-DFT/M06/Def2-TZVP level of theory (blue is the positive and orange means the negative densities).

Struct.	Natural Difference Orbitals		
C1	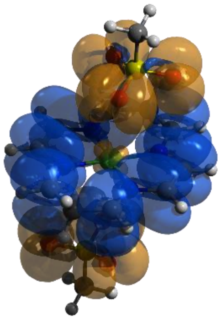	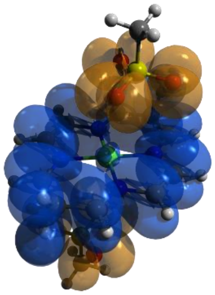	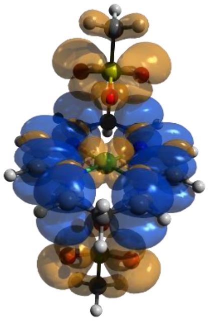
	S_6	S_{10}	S_{14}
	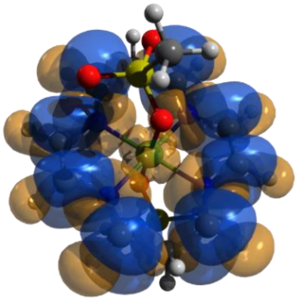	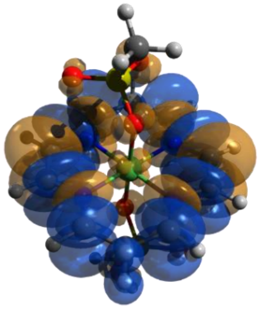	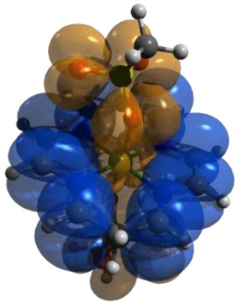
	T_4	T_8	T_{11}
C2	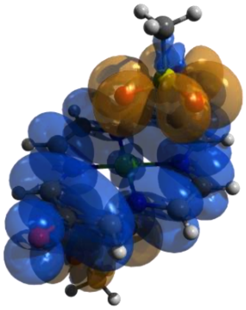	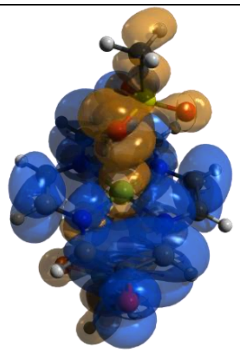	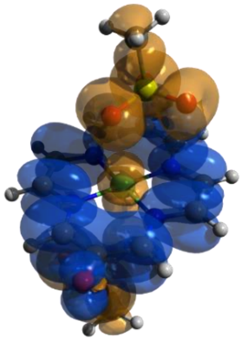
	S_4	S_8	S_{11}
	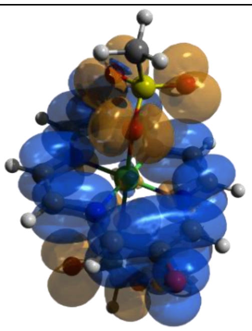	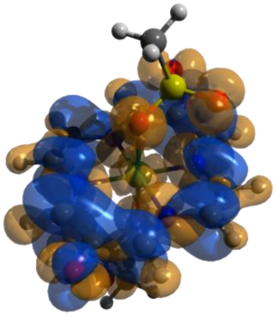	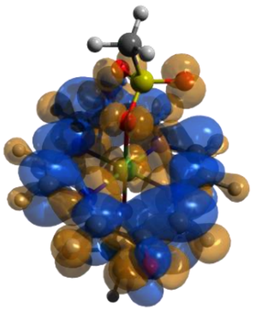
	T_4	T_8	T_{11}

C4

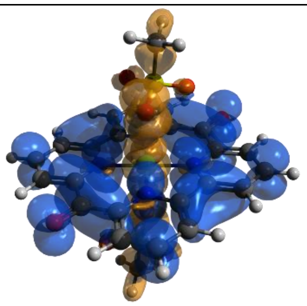

S_4

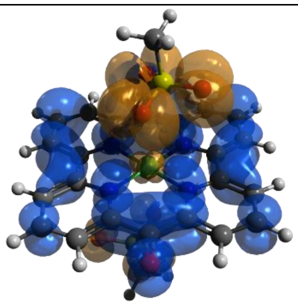

S_6

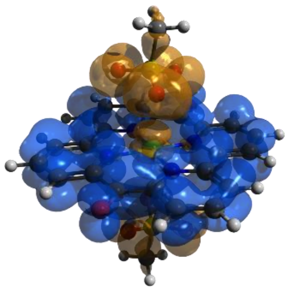

S_{10}

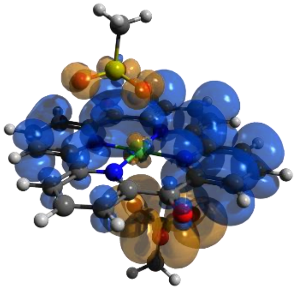

S_{14}

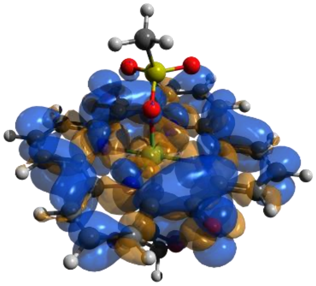

T_4

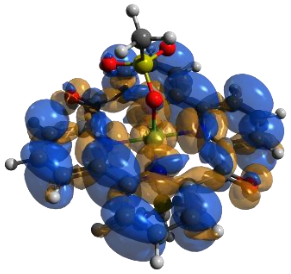

T_9

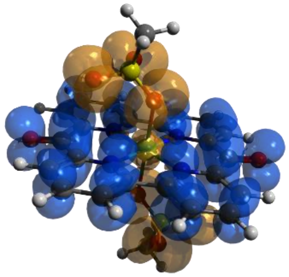

T_{13}

C5

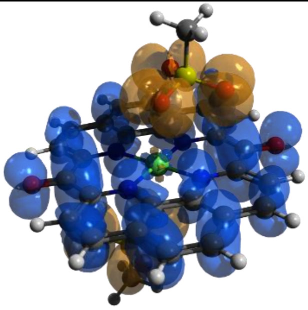

S_5

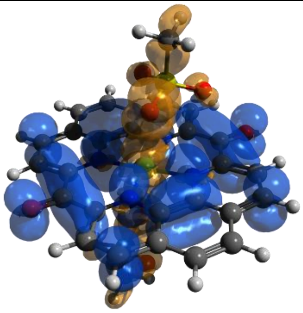

S_7

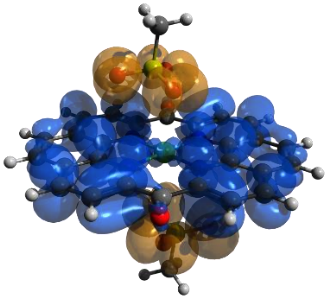

S_{12}

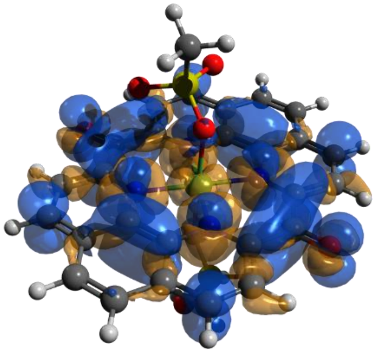

T_6

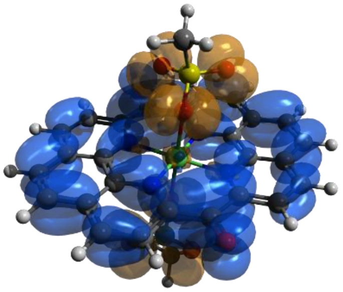

T_{12}

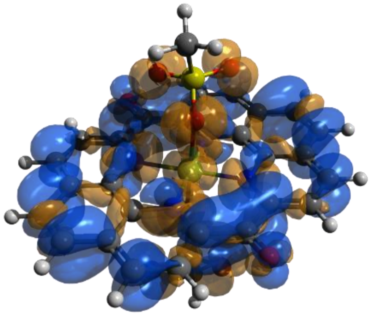

T_{14}

C6

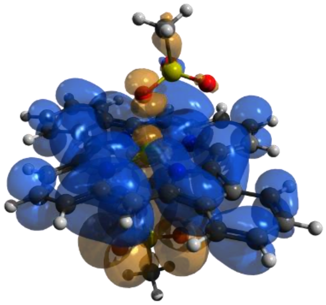

S_1

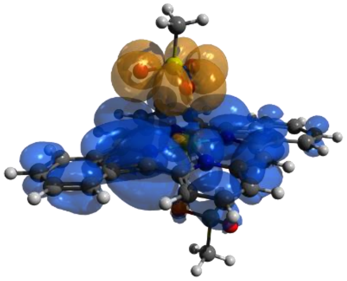

S_2

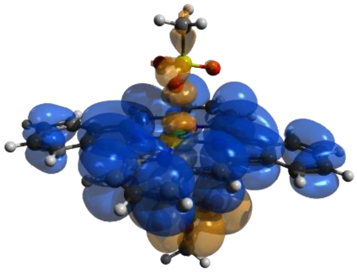

S_5

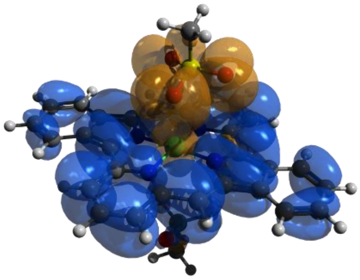

S_7

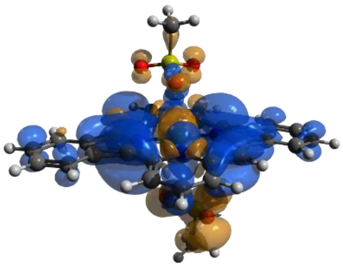

S_{10}

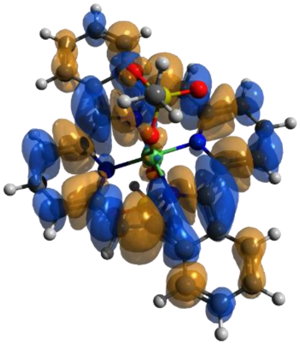

T_4

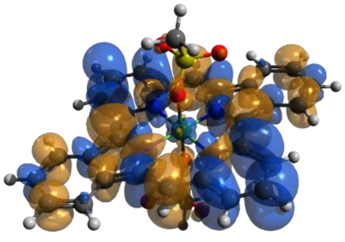

T_8

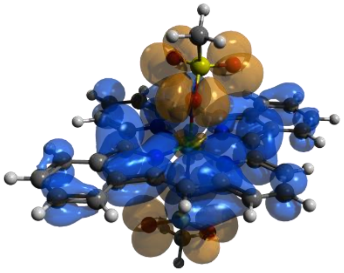

T_9

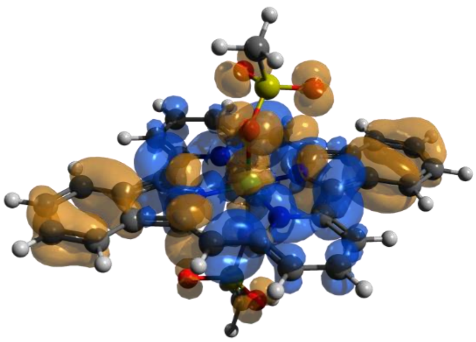

T_{15}

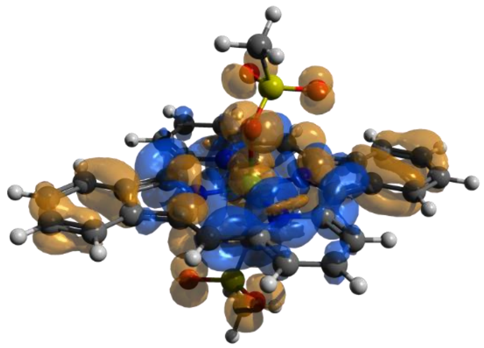

T_{16}

References

1. Zhao, Y.; Truhlar, D.G. A new local density functional for main-group thermochemistry, transition metal bonding, thermochemical kinetics, and noncovalent interactions. *J. Chem. Phys.* **2006**, *125*(19), 194101.
2. Wang, Y.; Jin, X.; Yu, H.S.; Truhlar, D.G.; Hea, X. Revised M06-L functional for improved accuracy on chemical reaction barrier heights, noncovalent interactions, and solid-state physics. *Proc. Natl. Acad. Sci. USA* **2017**, *114*(32), 8487–8492.
3. Salomon, O.; Reiher, M.; Hess, B. Assertion and validation of the performance of the B3LYP⋆ functional for the first transition metal row and the G2 test set. *J. Chem. Phys.* **2002**, *117*(10), 4729-4737.
4. Grimme, S.; Antony, J.; Ehrlich, S.; Krieg, S. A consistent and accurate ab initio parametrization of density functional dispersion correction (DFT-D) for the 94 elements H-Pu. *J. Chem. Phys.* **2010**, *132*, 154104.
5. Grimme, S.; Ehrlich, S.; Goerigk, L. Effect of the damping function in dispersion corrected density functional theory, *J. Comp. Chem.* **2011**, *32*(7), 1456-1465.
6. Zhao, Y.; Truhlar, D. The M06 suite of density functionals for main group thermochemistry, thermochemical kinetics, noncovalent interactions, excited states, and transition elements: two new functionals and systematic testing of four M06-class functionals and 12 other functionals. *Theor. Chem. Acc.* **2008**, *120*, 215-241.
7. Peverati, R.; Truhlar, D.G. Screened-exchange density functionals with broad accuracy for chemistry and solid-state physics. *Phys. Chem. Chem. Phys.* **2012**, *14*(47): 16187–16191.
8. Neese, F. The ORCA program system. *Wires Comput. Mol. Sci.* **2012**, *2*, 73-78.
9. Neese, F. Software update: the ORCA program system, version 4.0. *Wires Comput. Mol. Sci.* **2018**, *8*, e1327.
10. Weigend, F.; Ahlrichs, R. Balanced basis sets of split valence, triple zeta valence and quadruple zeta valence quality for H to Rn: Design and assessment of accuracy. *Phys. Chem. Chem. Phys.* **2005**, 7, 3297-3305.
11. GAUSSIAN09, Revision D.01, M. J. Frisch, G. W. Trucks, H. B. Schlegel, G. E. Scuseria, M. A. Robb et al. Gaussian, Inc., Wallingford CT, 2013.
